# Calculation of Homogenized Mechanical Coefficients of Fiber-Reinforced Composite Using Finite Element Method

**DOI:** 10.3390/ma17061334

**Published:** 2024-03-14

**Authors:** Mostafa Katouzian, Sorin Vlase, Calin Itu, Maria Luminita Scutaru

**Affiliations:** 1 Department of Mechanical Engineering, Transilvania University of Brașov, B-dul Eroilor, 20, 500036 Brașov, Romania; d-mec@unitbv.ro (M.K.); calinitu@unitbv.ro (C.I.); 2 Romanian Academy of Technical Sciences, B-dul Dacia, 26, 030167 Bucharest, Romania

**Keywords:** finite element method, FEM, reinforced composite, fiber, homogenized constants, epoxy matrix, generalized Hooke law, constitutive low

## Abstract

Determining the mechanical properties of a composite material represents an important stage in its design and is generally a complicated operation. These values are influenced by the topology and geometry of the resulting composite and the values of the elastic constants of the components. Due to the importance of this subject and the increasing use of composite materials, different calculation methods have been developed over the last fifty years. Some of the methods are theoretical, with results that are difficult to apply in practice due to difficulties related to numerical calculation. In the current paper, using theoretical results offered by the homogenization theory, values of engineering elastic constants are obtained. The finite element method (FEM) is used to determine the stress and strain field required in these calculations; this is an extremely powerful and verified calculation tool for the case of a material with any type of structure and geometry. In order to minimize errors, the paper proposes the method of least squares, a mathematical method that provides the best estimate for the set of values obtained by calculating FEM. It is useful to consider as many load cases as possible to obtain the best estimates. The elastic constants for a transversely isotropic material (composite reinforced with cylindrical fibers) are thus determined for a real case.

## 1. Introduction

Almost all industrial branches have had an explosive growth in the last few decades in the use of new and composite materials. It has been practically demonstrated that they represent in many cases a better alternative to the use of classic materials. This has determined, as a consequence, the development of manufacturing technologies for obtaining composite materials and structures made from these materials for industrial, medical, transport, military, aerospace, etc., applications. One of the major applications of composites has turned out to be structural applications. Structures made of composites have greater resistance and less weight. The existence of these materials has determined numerous studies that have dealt with the properties of these materials, their behavior in different conditions and the possibilities of improving properties needed for practical applications. A composite is composed of two or more phases of material, one playing the role of matrix and the other of reinforcing elements. Fiber-reinforced polymers stand out among matrix materials. The properties of these materials are defined by the matrix (usually polyester resin) and the reinforcing materials (fibers) [1].

In designing and manufacturing a structure that has composite materials in its composition, knowing the engineering constants of the materials is a major requirement. This necessity has led to numerous studies in this direction with the aim of providing the designer with efficient and fast calculation methods. This objective (knowing the mechanical properties of a composite) remains important in the current context of the continuous development of the use of composite materials. Up to now, a systematization of this research has not yet been carried out. Experimental measurements represent the safest method to obtain useful and credible results. Unfortunately, experimental methods are time-consuming and involve large expenses. As a result, the development of methods for the rapid estimation of elastic constants remains a current and continuous desire. There are a number of methods that will briefly be presented in the following. Most are focused on the study of linear behavior. But alongside these, improved methods have been developed that can analyze the non-linear behavior [2] of composites. Different mathematical principles are used. For example, a variational principle that also takes into account a time factor allows a relatively simple mathematical description of such cases [3,4,5]. The stress field induced by low temperatures in the body of a polymer composite at low temperatures (a spacecraft application is considered) is presented in [6], where the geometry of the composite microstructure is studied.

One of the methods used, in order to avoid the calculation of stresses and deformations in complex cases, is to consider particular loading cases [7]. Thus, in the mentioned paper, the material constants were calculated for an orthotropic composite and for an isotropic transverse composite. The disadvantage of this approach is that it provides lower and upper bounds for the values of the physical constants. Sometimes these methods can be somewhat or even totally useless because of the large range in which these values can be found [8,9,10,11]. Methods based on the micromechanical analysis of composites were among the first to be used [12,13]. Experimental results show good agreement with the results obtained using this method [4,5]. For the study of the most used composites, those consisting of a matrix and reinforced with fibers, numerous studies have been carried out, and the results have been presented in numerous papers published in recent years [14,15,16,17,18,19,20,21,22,23,24,25]. Other results related to the research of these types of problems are described in [26,27,28,29,30].

To analyze the visco-elastic response of a composite with short fibers, an analytical model is used in [31]. Thus, FEM proves to be a very useful tool in the case of determining the global mechanical properties of composite materials [32]. 

Special situations that may appear, such as the influence of temperature and humidity on the stress field for a unidirectional graphite/epoxy material, were also studied. These factors were found to lead to a decrease in the tensile strength of a silicon carbide unidirectional fiber composite. Brinson et al. used a similar model in [33,34,35] to determine material constants and how these values depend on the values of the component phase constants of a composite. In the present work, FEM analysis is presented to determine the stress and strain field in a transversely isotropic composite, in order to finally determine the values of the engineering constants for the studied material. Other studies concerning the behavior of these composites are presented in [36,37,38].

Micromechanical models used together with FEM to determine the stress and strain field were successfully used in [39] to determine the Young’s modulus and thermal expansion coefficient of a nanocomposite. For this type of approach, a three-dimensional volume-representative element with randomly distributed SiC reinforcement particles inside was used. Based on the results obtained from the calculation, conclusions that are useful in practical applications were formulated. The performed tests confirm the correctness of the performed calculation. A study of carbon fiber-reinforced polymer composites containing unwanted voids using FEM is presented in [40]. Voids will cause a strong decrease in the mechanical properties of a composite. The results of the study are in agreement with experimental measurements. A micromechanical model of intelligent composites reinforced with piezoelectric constituents is developed in [41]. A hybrid composite reinforced with carbon fibers based on carbon nanotubes where FEM is used for analysis is presented in [42]. The damping characteristics [43] of a unidirectional composite material reinforced with polymer fibers are determined in [44]. Again, micromechanical models show their efficiency in performing such calculations.

In the paper, the representative volume element (RVE) is used for microstructure analysis. This method lends itself well to the study of a large class of materials with very different topologies. Obviously, at the microstructure level, any composite material studied is a heterogeneous material. Its properties are determined by the structure of the composite and the properties of the component materials, matrix and reinforcement material. In the case of a finite element analysis model, these inhomogeneities are neglected. The properties of a single finite element are assumed to be constant.

The homogenized parameters of a composite that define the engineering constants of the material are determined based on a representative volume element using the finite element method. The obtained values agree very well with experimentally determined values. FEM also allows the analysis of some material variants, regarding the geometry and the percentage of the reinforcement material used. Other results concerning the application of FEM in the problems of determining the elastic constant of the composite material multiphasic are presented in [45,46,47,48,49,50,51]. In any of the proposed methods, FEM was always used to determine the field of stresses and deformations, which is used in different ways. Fiber-reinforced composites, used in a wide class of applications, have attracted the attention of researchers, and there are numerous studies on the subject. Some more interesting results obtained in the field can be mentioned. Thus, research has dealt with a wide class of materials, including cement [52]. FEM was used to obtain a field of stresses and strains, which was then used with homogenization theory to determine two material constants for cement reinforced with metal fibers. The obtained values proved to be in accordance with the values obtained using other calculation methods. Polymeric composites reinforced with natural fibers are used in many applications at the moment. A study that aims to determine the visco/elastic constants of these materials is presented in [53]. Natural fibers have viscoelastic behavior and superior specific properties. They also have good ecological characteristics and a low cost. The mentioned work proposes an analytical model of homogenization. Experimental checks demonstrate the accuracy of the model used. A method for determining the mechanical properties of composites reinforced with fibers (carbon and glass) is presented in [54]. This type of material has viscoplastic behavior. The creep behavior of such a material subjected to high temperatures is determined using the presented theory and is verified experimentally. Other research in the field for different situations that can be encountered in practice is presented in [55,56].

It is expected that the calculation accuracy is that normally obtained by applying FEM. In general, FEM has proven to be a reliable method that provides credible and sufficiently accurate results for engineering applications.

In this research, based on micromechanical models, the authors used FEM to calculate the mechanical constants of a composite material reinforced with unidirectional cylindrical fibers. Results are obtained for a composite in which the epoxy matrix is reinforced with carbon fibers. The method can be applied to any type of composite reinforced with fibers, if as a whole it can be considered as a homogeneous transversely isotropic body. In the paper, the authors considered several loading cases for the studied specimen, thus obtaining more information regarding the elastic behavior of the material. In order to obtain the best possible result, the least squares method was applied. Thus, an objective criterion for determining the elastic constants is provided. It can be specified that these elastic constants are obtained and no upper or lower bounds are obtained for them, as happens in a series of energetic methods, previously presented.

The presented method is extremely suitable for the design phase, where it is desirable to know sufficiently precise estimates of the mechanical properties of a material in order to create a mechanical structure. Obviously, after the adoption of a solution and a project and the realization of a prototype, experimental measurements can be made to provide numerical values for the estimated properties that allow possible corrections of the project (if it proves necessary).

The estimation method proposed in the paper is based on models previously established by researchers, which establish formula relationships that can be used to help to determine the mechanical properties of a material if the topology, geometry and properties of the component phases are taken into account. Most of the methods are definitively based on the determination of the field of stress and strain for certain load states of the material. This can be achieved, in most cases, only using numerical methods, which are approximate methods. During the work, the least squares method was proposed to find the best possible approximation, based on the calculation of the stress–strain field in a composite body in several loading situations. Thus, a theoretical basis is provided for this type of estimation. 

Models used up to now deal with the simulation of particular loading cases on the basis of which one of the elastic constants can be calculated. The disadvantage of such analysis is that the models generally provide upper and lower limits for elastic constants, which are often very imprecise (the given interval is very large). Other methods, such as the homogenization method, imply as a preliminary stage knowledge of the stress and strain field, a difficult thing to calculate in general, which leads to the use of numerical calculation methods, implicitly FEM. After determining these values, quite difficult calculations follow to obtain these coefficients. The least squares method offers a more direct approach; the method is well known, and the consideration of a larger number of analyzed cases leads to more accurate values of the elastic constants with the help of the least squares criterion.

## 2. Materials and Methods

**Figure 1:**
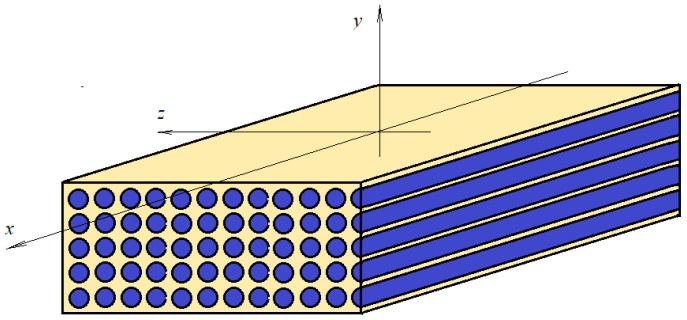
Composite material: transversally isotropic.

Let us consider a composite material made of an epoxy matrix reinforced with aligned cylindrical fibers (Figure 1). The orientation of the fibers defines the axis of symmetry. If a reference frame is considered, as in Figure 1, the properties along the x direction are different if the properties along the y and z directions are considered. In the y and z directions, the properties are the same. 

Plane *yz* is an isotropic plane. The properties of such a material are defined by five parameters (the longitudinal (axial) Young’s modulus *E_A_*, the transverse Young’s modulus *E_T_*, the transverse and longitudinal Poisson’s ratio (ν_A_ and ν_T_) and the shear modulus *G_A_*).

The composite material as a whole is treated as a transversely isotropic material. The properties that are used in strength calculations represent the global properties of the composite, which is composed of two phases (fiber and matrix) that have different properties. Obviously, practical applications require knowledge of the global properties of the material. Starting from the individual properties of the two phases, it is necessary to determine the properties of the material. Various calculation methods have been used to determine them. The first methods applied were methods that used micromechanical models [10,11,12,13]. However, these led to the need to know the field of stress and strain in the resulting material and to obtain the average of the values for stresses and strains. In general, this method, which involves numerical calculations, was used to determine only some engineering constants, simply obtained through special loading states [1]. Variational methods also require the knowledge of functions that define the stress and deformation field. Other calculation methods [1] operate with simplifications of the model and approximations for the performed calculations. In general, in most of the works that deal with these problems, lower and higher bounds for engineering constants (buck modulus, Young’s moduli and Poisson’s ratios) are determined. Although useful, for certain areas of fiber concentration (generally towards the middle of the range), the errors can be huge. This requires the use of methods that bring us closer to the real values.

In this work, the authors proposed to use a micromechanical model for an elementary cell (which takes into account a single fiber of a given length embedded in a matrix). This elementary cell is an RVE, and the material in the assembly can be conceived as an overlap of such RVEs. Using the results from micromechanical analysis to determine the equivalent engineering constants of the RVE, it is necessary to determine the stress and strain field for an arbitrary load and then to determine the average of these quantities [1]. Considering fundamental relationships in the mechanics of transversely isotropic continuous media, the values of the equivalent engineering constants can then be determined.

**Figure 2:**
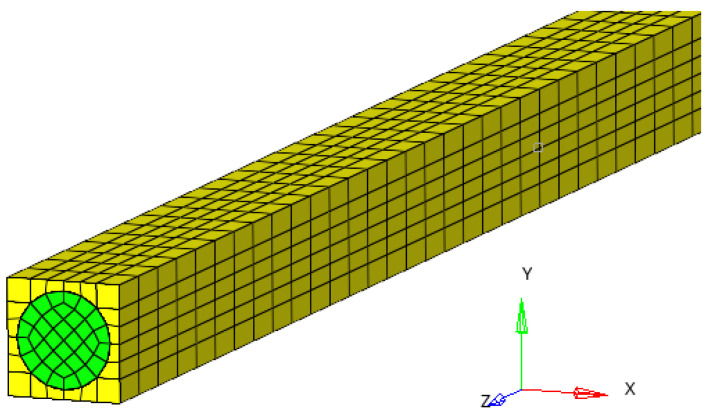
The RVE for finite element analysis.

The study is carried out for an RVE and for a package of elementary cells (Figure 2).

Equations that provide the relationship between stresses and strains, assuming isothermal conditions, considering a transversely isotropic material, are as follows [13]:

(1) $$\left\{\begin{array}{c} {ο}_{xx}\\ {ο}_{yy}\\ {ο}_{zz}\\ {ο}_{yz}\\ {ο}_{zx}\\ {ο}_{xy} \end{array}\right\}=\left[\begin{array}{cccccc} C_{11} & C_{12} & C_{12} & 0 & 0 & 0\\ C_{12} & C_{22} & C_{23} & 0 & 0 & 0\\ C_{12} & C_{23} & C_{22} & 0 & 0 & 0\\ 0 & 0 & 0 & C_{44} & 0 & 0\\ 0 & 0 & 0 & 0 & C_{66} & 0\\ 0 & 0 & 0 & 0 & 0 & C_{66} \end{array}\right]\left\{\begin{array}{c} \varepsilon_{xx}\\ \varepsilon_{yy}\\ \varepsilon_{zz}\\ 2\varepsilon_{yz}\\ 2\varepsilon_{zx}\\ 2\varepsilon_{xy} \end{array}\right\}$$

where:

(2) $$C_{44}=\frac{C_{22}-C_{23}}{2}$$

or:

(3) $$\left\{\sigma \right\}=\left[C\right]\left\{\varepsilon \right\},$$

Considering microstructural models [14,15], it can be considered that any part of the composite behaves as a homogeneous transversely isotropic body [57,58].

As a result, it is considered that if an RVE is taken, its behavior to any demands is, on average, identical to the behavior of the entire isotropic transverse body. In essence, the stress and strain field for an RVE is calculated, averages of the stress and strain values for the entire RVE are calculated, and then it is considered that these values must comply with Hooke’s generalized law for transversely isotropic materials. In this way, a set of equations is obtained that connects the average stress and strain to the material constants. The obtained set of equations is used to determine these constants.

Obviously, FEM, representing an approximation method, introduces errors into calculations made. For this reason, a single calculation of the requests that appear in the RVE could bring errors in the determination of the material constants. For this reason, a large number of calculations are made with different types of load, after which the least square method is used to determine the best approximation for the material constants.

In the following, the average of a size will be denoted with a bar over the symbol of the size.

With FEM, the values of stresses and strains are determined numerically for an RVE consisting of a fiber incorporated in a matrix. A case of loading the RVE is considered. If $n_{f}$ is the number of finite elements through which the fiber that is included in the RVE was discretized, $n_{m}$ is the number of finite elements in which the matrix was discretized and $n_{t}=n_{f}+n_{m}$ is the total number of finite elements; after FEM modeling, sets of equations with stress values and strains ${\left\{\sigma \right\}}_{k},\;{\left\{\varepsilon \right\}}_{k};\;k=\bar{1,n_{f}}$, ${\left\{\sigma \right\}}_{l},\;{\left\{\varepsilon \right\}}_{l};\;l=\bar{1,n_{m}}$ are used for each considered finite element. Vectors $\left\{\sigma \right\}$ and $\left\{\varepsilon \right\}$ have the following components:

(4) $$\left\{\sigma \right\}=\left\{\begin{array}{c} \sigma_{xx}\\ \sigma_{yy}\\ \sigma_{zz}\\ \sigma_{yz}\\ \sigma_{zx}\\ \sigma_{xy} \end{array}\right\};\;\left\{\varepsilon \right\}=\left\{\begin{array}{c} \varepsilon_{xx}\\ \varepsilon_{yy}\\ \varepsilon_{zz}\\ \gamma_{yz}\\ \gamma_{zx}\\ \gamma_{xy} \end{array}\right\}=\left\{\begin{array}{c} \varepsilon_{xx}\\ \varepsilon_{yy}\\ \varepsilon_{zz}\\ 2\varepsilon_{yz}\\ 2\varepsilon_{zx}\\ 2\varepsilon_{xy} \end{array}\right\}.$$

Averages of these sizes for the entire RVE are given by:

(5) $$\left\{\bar{\sigma }\right\}=\nu_{f}{\left\{\bar{\sigma }\right\}}^{\left(f\right)}+\nu_{m}{\left\{\bar{\sigma }\right\}}^{\left(m\right)};$$

(6) $$\left\{\bar{\varepsilon }\right\}=\nu_{f}{\left\{\bar{\varepsilon }\right\}}^{\left(f\right)}+\nu_{m}{\left\{\bar{\varepsilon }\right\}}^{\left(m\right)},$$

where index (*f*) is used to denote a property for a fiber and index (*m*) is used for a matrix. The average stresses in the fiber and matrix, respectively, are:

(7) $${\left\{\bar{\sigma }\right\}}^{\left(f\right)}=\frac{\sum_{i=1}^{n_{f}}\sigma_{i}^{\left(f\right)}}{n_{f}};\;{\left\{\bar{\sigma }\right\}}^{\left(m\right)}=\frac{\sum_{i=1}^{n_{m}}\sigma_{i}^{\left(m\right)}}{n_{m}},$$

and the average strains in the fiber and matrix, respectively, are:

(8) $${\left\{\bar{\varepsilon }\right\}}^{\left(f\right)}=\frac{\sum_{i=1}^{n_{f}}\varepsilon_{i}^{\left(f\right)}}{n_{f}};\;{\left\{\bar{\varepsilon }\right\}}^{\left(m\right)}=\frac{\sum_{i=1}^{n_{m}}\varepsilon_{i}^{\left(m\right)}}{n_{m}}.$$

Here, $\sigma_{i}^{\left(f\right)}$, $\sigma_{i}^{\left(m\right)}$, $\varepsilon_{i}^{\left(f\right)}$, $\varepsilon_{i}^{\left(m\right)}$ represent the vectors of stress and strain in the RVE, labelled with index i for fiber and matrix, respectively. Compared to the exact theoretical value that exists when considering the RVE as an isotropic transverse body, there is a deviation that can be written as follows:

(9) $$\left\{\delta \right\}=\left\{\bar{\sigma }\right\}-\left[C\right]\left\{\bar{\varepsilon }\right\},$$

with components:

(10) $$\delta_{1}={\bar{\sigma }}_{xx}-C_{11}{\bar{\varepsilon }}_{xx}-C_{12}\left({\bar{\varepsilon }}_{yy}+{\hat{\varepsilon }}_{zz}\right),$$

(11) $$\delta_{2}={\bar{\sigma }}_{yy}-C_{12}{\bar{\varepsilon }}_{xx}-C_{22}{\bar{\varepsilon }}_{yy}-C_{23}{\bar{\varepsilon }}_{zz},$$

(12) $$\delta_{3}={\bar{\sigma }}_{zz}-C_{12}{\bar{\varepsilon }}_{xx}-C_{23}{\bar{\varepsilon }}_{yy}-C_{22}{\bar{\varepsilon }}_{zz},$$

(13) $$\delta_{4}={\bar{\sigma }}_{yz}-\left(C_{22}-C_{23}\right){\bar{\varepsilon }}_{yz},$$

(14) $$\delta_{5}={\bar{\sigma }}_{zx}-2C_{66}{\bar{\varepsilon }}_{zx},$$

(15) $$\delta_{6}={\dot{\sigma }}_{xy}-2C_{66}{\bar{\varepsilon }}_{xy},$$

The total squared deviation for the considered load case is

(16) $$\left\{\delta^{2}\right\}={\left\{\delta \right\}}^{T}\left\{\delta \right\}={\left\{\bar{\sigma }\right\}}^{T}\left\{\bar{\sigma }\right\}-{\left\{\bar{\sigma }\right\}}^{T}\left[C\right]\left\{\bar{\varepsilon }\right\}-{\left\{\bar{\varepsilon }\right\}}^{T}{\left[C\right]}^{T}\left\{\bar{\sigma }\right\}+{\left\{\bar{\varepsilon }\right\}}^{T}{\left[C\right]}^{T}\left[C\right]\left\{\bar{\varepsilon }\right\},$$

Let us consider that the FEM calculation is now conducted for a particular case of loading. Let us index with *i* the considered case. In the case under study, different loading cases will be considered, so $i=\bar{1,n}$. In this case, the components of the deviation vector for the considered state of load will be noted as f:

(17) $$\delta_{1}(i)={\bar{\sigma }}_{xx,i}-C_{11}{\bar{\varepsilon }}_{xx,i}-C_{12}\left({\bar{\varepsilon }}_{yy,i}+{\bar{\varepsilon }}_{zz,i}\right),$$

(18) $$\delta_{2}(i)={\bar{\sigma }}_{yy,i}-C_{12}{\bar{\varepsilon }}_{xx,i}-C_{22}{\bar{\varepsilon }}_{yy,i}-C_{23}{\bar{\varepsilon }}_{zz,i},$$

(19) $$\delta_{3}(i)={\bar{\sigma }}_{zz,i}-C_{12}{\bar{\varepsilon }}_{xx,i}-C_{23}{\bar{\varepsilon }}_{yy,i}-C_{22}{\bar{\varepsilon }}_{zz,i},$$

(20) $$\delta_{4}(i)={\bar{\sigma }}_{yz,i}-\left(C_{22}-C_{23}\right){\bar{\varepsilon }}_{yz,i},$$

(21) $$\delta_{5}(i)={\bar{\sigma }}_{zx,i}-2C_{66}{\bar{\varepsilon }}_{zx,i},$$

(22) $$\delta_{6}(i)={\bar{\sigma }}_{xy,i}-2C_{66}{\bar{\varepsilon }}_{xy,i},$$

The squared deviation for the loading state labelled *i* is:

(23) $$\begin{array}{c} \delta^{2}(i)=\delta_{1}^{2}(i)+\delta_{2}^{2}(i)+\delta_{3}^{2}(i)+\delta_{4}^{2}(i)+\delta_{5}^{2}(i)+\delta_{6}^{2}(i)\\ ={\left[{\bar{\sigma }}_{xx,i}-C_{11}{\bar{\varepsilon }}_{xx,i}-C_{12}\left({\bar{\varepsilon }}_{yy,i}+{\bar{\varepsilon }}_{zz,i}\right)\right]}^{2}+{\left[{\bar{\sigma }}_{yy,i}-C_{12}{\bar{\varepsilon }}_{xx,i}-C_{22}{\bar{\varepsilon }}_{yy,i}-C_{23}{\bar{\varepsilon }}_{zz,i}\right]}^{2}\\ +{\left[{\bar{\sigma }}_{zz,i}-C_{12}{\bar{\varepsilon }}_{xx,i}-C_{23}{\bar{\varepsilon }}_{yy,i}-C_{22}{\bar{\varepsilon }}_{zz,i}\right]}^{2}+{\left[{\bar{\sigma }}_{yz,i}-\left(C_{22}+C_{23}\right){\bar{\varepsilon }}_{yz,i}\right]}^{2}\\ +{\left[{\bar{\sigma }}_{zx,i}-2C_{66}{\bar{\varepsilon }}_{zx,i}\right]}^{2}+{\left[{\bar{\sigma }}_{xy,i}-2C_{66}{\bar{\varepsilon }}_{xy,i}\right]}^{2} \end{array}$$

The total squared deviation is:

(24) $$\begin{array}{c} \delta^{2}=\sum_{i=1}^{n}\delta {\left(i\right)}^{2}=\sum_{i=1}^{n}{\left[{\bar{\sigma }}_{xx,i}-C_{11}{\bar{\varepsilon }}_{xx,i}-C_{12}\left({\bar{\varepsilon }}_{yy,i}+{\bar{\varepsilon }}_{zz,i}\right)\right]}^{2}\\ +\sum_{i=1}^{n}{\left[{\bar{\sigma }}_{yy,i}-C_{12}{\bar{\varepsilon }}_{xx,i}-C_{22}{\bar{\varepsilon }}_{yy,i}-C_{23}{\bar{\varepsilon }}_{zz,i}\right]}^{2}\\ +\sum_{1}^{n}{\left[{\bar{\sigma }}_{zz,i}-C_{12}{\bar{\varepsilon }}_{xx,i}-C_{23}{\bar{\varepsilon }}_{yy,i}-C_{22}{\bar{\varepsilon }}_{zz,i}\right]}^{2}+\sum_{1}^{n}{\left[{\bar{\sigma }}_{yz,i}-\left(C_{22}+C_{23}\right){\bar{\varepsilon }}_{yz,i}\right]}^{2}\\ +\sum_{1}^{n}{\left[{\bar{\sigma }}_{zx,i}-2C_{66}{\bar{\varepsilon }}_{zx,i}\right]}^{2}+\sum_{1}^{n}{\left[{\bar{\sigma }}_{xy,i}-2C_{66}{\bar{\varepsilon }}_{xy,i}\right]}^{2}. \end{array}$$

The condition for this squared deviation to be minimal is given by relationships offered by mathematical analysis:

(25) $$\frac{\partial \delta^{2}}{\partial C_{11}}=-2\left[\sum_{i=1}^{n}{\bar{\sigma }}_{xx,i}{\bar{\varepsilon }}_{xx,i}-C_{11}\sum_{i=1}^{n}{\bar{\varepsilon }}_{xx,i}^{2}-C_{12}\left(\sum_{i=1}^{n}{\bar{\varepsilon }}_{yy,i}{\bar{\varepsilon }}_{xx,i}+\sum_{i=1}^{n}{\bar{\varepsilon }}_{zz,i}{\bar{\varepsilon }}_{xx,i}\right)\right]=0,$$

(26) $$\begin{array}{c} \frac{\partial \delta^{2}}{\partial C_{12}}=-2\left[\sum_{i=1}^{n}{\bar{\sigma }}_{xx,i}\left({\bar{\varepsilon }}_{yy,i}+{\bar{\varepsilon }}_{zz,i}\right)-C_{11}\sum_{i=1}^{n}{\bar{\varepsilon }}_{xx,i}\left({\bar{\varepsilon }}_{yy,i}+{\bar{\varepsilon }}_{zz,i}\right)-C_{12}\sum_{i=1}^{n}{\left({\bar{\varepsilon }}_{yy,i}+{\bar{\varepsilon }}_{zz,i}\right)}^{2}\right]\\ -2\left[\sum_{1}^{n}{\bar{\sigma }}_{yy,i}{\bar{\varepsilon }}_{xx,i}-C_{12}\sum_{1}^{n}{\bar{\varepsilon }}_{xx,i}^{2}-C_{22}\sum_{1}^{n}{\bar{\varepsilon }}_{xx,i}{\bar{\varepsilon }}_{yy,i}-C_{23}\sum_{1}^{n}{\bar{\varepsilon }}_{xx,i}{\bar{\varepsilon }}_{zz,i}\right]\\ -2\left[\sum_{1}^{n}{\bar{\sigma }}_{zz,i}{\bar{\varepsilon }}_{xx,i}-C_{12}\sum_{1}^{n}{\bar{\varepsilon }}_{xx,i}^{2}-C_{23}\sum_{1}^{n}{\bar{\varepsilon }}_{xx,i}{\bar{\varepsilon }}_{yy,i}-C_{22}\sum_{1}^{n}{\bar{\varepsilon }}_{xx,i}{\bar{\varepsilon }}_{zz,i}\right]=0, \end{array}$$

(27) $$\begin{array}{c} \frac{\partial \delta^{2}}{\partial C_{22}}=-2{\left[\sum_{i}^{n}{\bar{\sigma }}_{yy,i}{\bar{\varepsilon }}_{yy,i}-C_{12}\sum_{1}^{2}{\bar{\varepsilon }}_{xx,i}{\bar{\varepsilon }}_{yy,i}-C_{22}\sum_{1}^{n}{\bar{\varepsilon }}_{yy,1}^{2}-C_{23}\sum_{1}^{n}{\bar{\varepsilon }}_{yy,i}{\bar{\varepsilon }}_{zz,i}\right]}^{2}\\ -2\left[\sum_{1}^{n}{\bar{\sigma }}_{zz,i}{\bar{\varepsilon }}_{zz,i}-C_{12}\sum_{1}^{n}{\bar{\varepsilon }}_{xx,i}{\bar{\varepsilon }}_{zz,i}-C_{23}\sum_{1}^{n}{\bar{\varepsilon }}_{yy,i}{\bar{\varepsilon }}_{zz,i}-C_{22}\sum_{1}^{n}{\hat{\varepsilon }}_{zz,i}^{2}\right]\\ -2\left[\sum_{1}^{n}{\bar{\sigma }}_{yz,i}{\bar{\varepsilon }}_{yz,i}-\left(C_{22}+C_{23}\right)\sum_{1}^{n}{\bar{\varepsilon }}_{yz,i}^{2}\right]=0, \end{array}$$

(28) $$\begin{array}{c} \frac{\partial \delta^{2}}{\partial C_{23}}=-2\left[\sum_{1}^{n}{\bar{\sigma }}_{yy,i}{\bar{\varepsilon }}_{zz,i}-C_{12}\sum_{i}^{n}{\bar{\varepsilon }}_{xx,i}{\bar{\varepsilon }}_{zz,i}-C_{22}\sum_{1}^{n}{\bar{\varepsilon }}_{yy,i}{\bar{\varepsilon }}_{zz,i}-C_{23}\sum_{1}^{n}{\bar{\varepsilon }}_{zz,i}^{2}\right]\\ -2\left[\sum_{1}^{n}{\bar{\sigma }}_{zz,i}{\bar{\varepsilon }}_{yy,i}-C_{12}\sum_{1}^{n}{\bar{\varepsilon }}_{xx,i}{\bar{\varepsilon }}_{yy,i}-C_{23}\sum_{1}^{n}{\bar{\varepsilon }}_{yy,i}^{2}-C_{22}\sum_{1}^{n}{\bar{\varepsilon }}_{zz,i}{\bar{\varepsilon }}_{yy,i}\right]\\ -2\left[\sum_{1}^{n}{\bar{\sigma }}_{yz,i}{\bar{\varepsilon }}_{yz,i}-\left(C_{22}+C_{23}\right)\sum_{1}^{n}{\bar{\varepsilon }}_{yz,i}^{2}\right]=0, \end{array}$$

(29) $$\frac{\partial \delta^{2}}{\partial C_{66}}=-2\left[\sum_{1}^{n}{\bar{\sigma }}_{zx,i}{\bar{\varepsilon }}_{zx,i}-2C_{66}\sum_{i}^{n}{\bar{\varepsilon }}_{zx,i}^{2}\right]-2\left[\sum_{1}^{n}{\bar{\sigma }}_{xy,i}{\bar{\varepsilon }}_{xy,i}-2C_{66}\sum_{i}^{n}{\bar{\varepsilon }}_{xy,i}^{2}\right]=0,$$

The system obtained by applying these conditions represents a linear system of five equations with five unknowns. By solving it, the five unknowns can be obtained, which represent material constants with the help of which the usual elastic engineering constants can then be determined: Young’s moduli, shear moduli, bulk modulus, Poisson’s ratios, etc.

Equation (29) immediately results in the following:

(30) $$C_{66}=\frac{\sum_{1}^{n}{\bar{\sigma }}_{zx,i}{\bar{\varepsilon }}_{zx,i}+\sum_{1}^{n}{\bar{\sigma }}_{xy,i}{\bar{\varepsilon }}_{xy,i}}{2\left(\sum_{i}^{n}{\bar{\varepsilon }}_{zx,i}^{2}+\sum_{i}^{n}{\bar{\varepsilon }}_{xy,i}^{2}\right)},$$

The system remaining to be solved is presented in Appendix A and offers other constants of materials, $C_{11},C_{12},C_{22},C_{23}$. Using these values now, it is possible to obtain the desired engineering elastic coefficients.

Bulk modulus can be computed by the following relationship:

(31) $$K_{23}=C_{22},$$

The shear modulus is:

(32) $$G_{23}=\frac{C_{22}-C_{23}}{2}=C_{66}=\frac{{\bar{\sigma }}_{22}-{\bar{\sigma }}_{33}}{2\left({\bar{\varepsilon }}_{22}-{\bar{\varepsilon }}_{33}\right)}=\frac{{\bar{\sigma }}_{23}}{2{\bar{\varepsilon }}_{23}}.$$

Other relationships between the elastic coefficients give us all the values that are necessary for the resistance calculation of a structure made of the proposed material.

(33) $$\nu_{1}=\nu_{21}=\nu_{31}=\frac{1}{2}{\left(\frac{C_{11}-E_{11}}{K_{23}}\right)}^{1/2}=\frac{C_{12}}{C_{22}+C_{33}};$$

(34) $$\psi =1+\frac{4\nu_{1}^{2}K_{23}}{E_{11}};$$

(35) $$E_{22}=E_{33}=\frac{4G_{23}K_{23}}{K_{23}+\psi G_{23}};$$

(36) $$\nu_{23}=\frac{K_{23}-\psi G_{23}}{K_{23}+\psi G_{23}};$$

(37) $$C_{66}=\frac{{\bar{\sigma }}_{12}}{2{\bar{\varepsilon }}_{12}}=\frac{{\bar{\tau }}_{12}}{{\bar{\gamma }}_{12}}=G_{12}=G_{13}=G_{1}.$$

## 3. Results

A composite bar made up of an epoxy matrix reinforced with a circular fiber is considered. In this way, a rectangular parallelepiped is obtained, which represents an RVE. The results obtained for this RVE will be used in the calculations. In a perfected version, a rectangular parallelepiped made up of 16 such RVEs is considered. In this case, a calculation can be made more precisely. To see the potential application of the proposed method, a micromechanical FEM model is developed for a composite bar made of a glass/epoxy matrix reinforced with carbon fibers [59].

Values of the main material properties for the two phases of the composite material are:

*E_m_ =* 4.14 *GPa*; *ν_m_ =* 0.22; *E_f_ =* 86.90 *GPa*; *ν_f_ =* 0.34.

The two materials are considered homogeneous and isotropic. They are defined by two material constants. For the situation studied in the paper, the fiber volume ration of the total volume is $v_{f}=54.54\%$ and represents an important parameter in all methods of homogenization.

**Figure 3:**
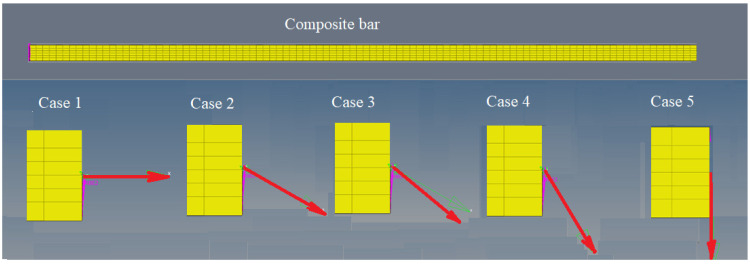
Position of force in the five cases of loading.

Five distinct load cases of the bar are considered (Figure 3). The load is considered to be a force that acts on one of the ends of the bar, the other end being embedded. In the five cases, the force acts under angles equal to 0, 30°, 45°, 60° and 90° from the x axis. For each of these cases, the stress and strain field is calculated and then the average of these stresses and strain is calculated for the entire RVE. Based on these values, elastic constants are then determined.

In the framework of analysis carried out in the work, the stresses and deformations are determined with the help of Altair software 2020 and the Hypermesh preprocessor and Hyperview postprocessor, respectively. These values are then used to determine the average values of stresses and strains for the fiber, the matrix and then for the RVE. Table 1, Table 2, Table 3, Table 4, Table 5, Table 6 and Table 7 present the results of the analysis.

**Figure 4:**
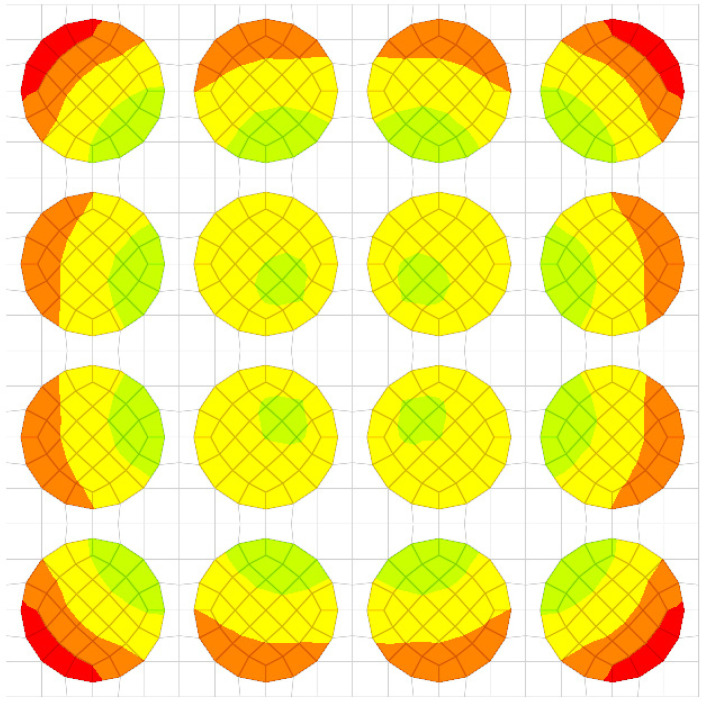
The elementary cell made up of 16 fibers and a qualitative representation of displacements (in colors).

The same calculations were also carried out considering a material cell made up of 16 fibers (Figure 4).

**Figure 5:**
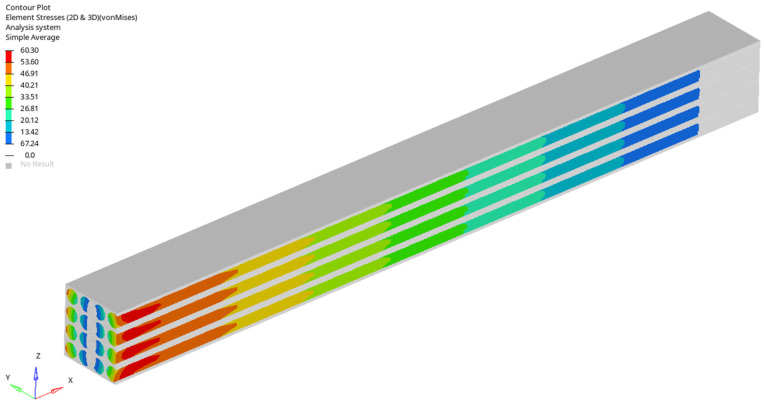
Stress distribution for second loading case (in GPa).

**Figure 6:**
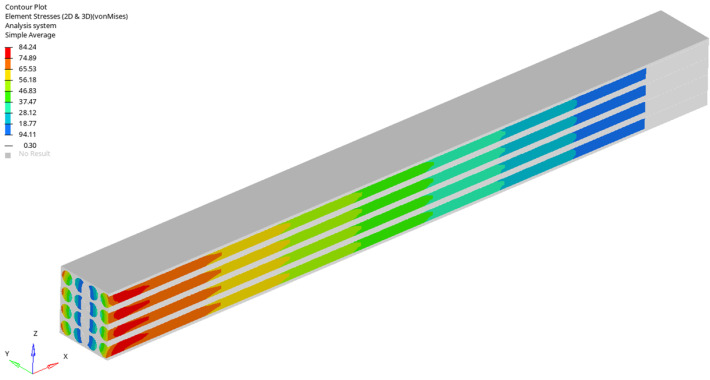
Stress distribution for third loading case (in GPa).

**Figure 7:**
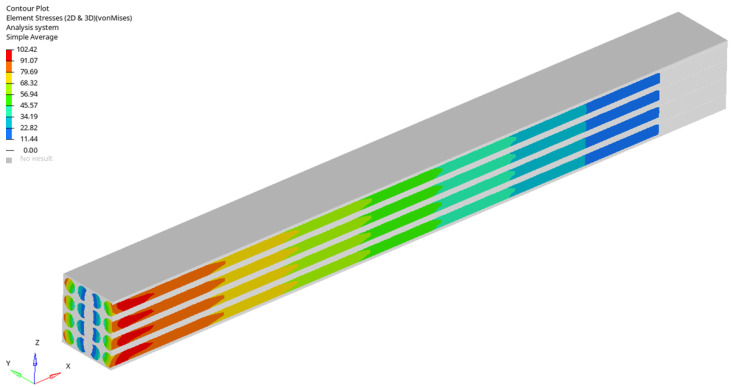
Stress distribution for fourth loading case (in GPa).

**Figure 8:**
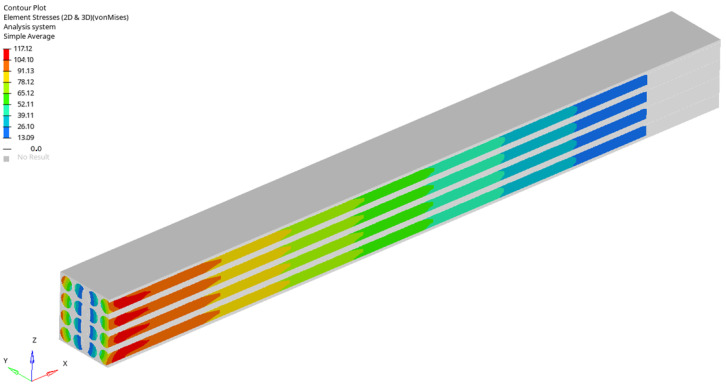
Stress distribution for fifth loading case.

**Figure 9:**
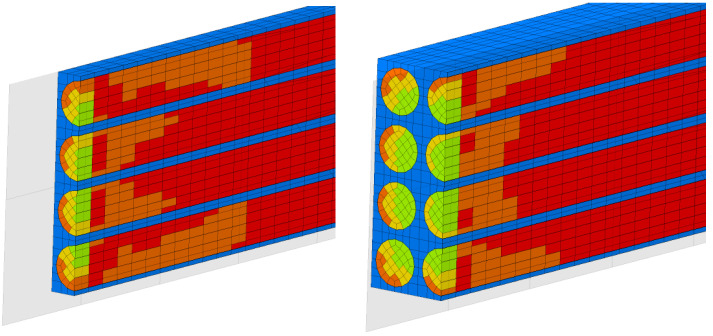
Sections of the first and second rows. Colored are a qualitative representation of the displacements.

**Figure 10:**
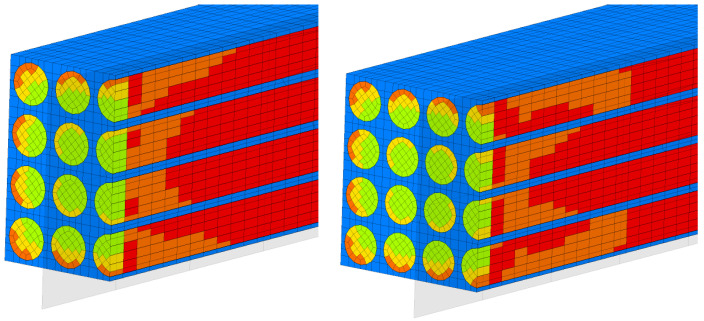
Sections of the third and fourth rows. Colored are a qualitative representation of the displacements.

For a load case, the stresses in the sections made in the first, second, third and fourth rows are represented (Figure 5, Figure 6, Figure 7 and Figure 8). It is observed that these tensions vary quite strongly at the ends, unrelated to the overall stress of the bar. This occurs at the ends of the bar: both the loaded end of the bar and the recessed end (Figure 9 and Figure 10). Due to these variations, when the averages were calculated, the values obtained in this part of the bar were removed. In the calculations made in the study, 200 finite elements were removed from one end and 200 finite elements were removed from the other end.

## 4. Discussion

The theoretical models developed by the researchers presented in the introduction section allow the determination of the elastic constants of a material considering a single RVE. A linear system of five equations with five unknowns is obtained that provides these values. However, these values are obtained for a single loading case of the material, based on the stress and deformation field obtained for that loading, with errors arising from the determination of this field. The method used is FEM, which, being an approximation method, inherently introduces some errors. The obtained constants will be affected by these errors. If different loading situations are considered, the errors will be different and, obviously, will depend on these loading cases. To minimize the importance of these errors in the calculation of the elastic constants, several load cases will be considered and the condition is set to look for those values that minimize the quadratic deviation. It is obvious that a large number of considered load cases will minimize the errors that may occur in some situations where the errors may be too large. It is therefore recommended to consider very different loading situations, so as to cover as wide a range of situations as possible. In the case of the present study, these were considered as five distinct load cases. 

Most of the methods used to determine the properties of a composite material obtain results in the form of inequalities, which provide the sought constants in a certain interval. For some concentrations of the phases, these ranges may be wide enough so that the information provided is ineffective. The presented method provides values that are affected only by the errors involved in the FEM. This leads to much more useful results for the designer. Experimental checks for simpler situations, where only a single loading state is considered, are presented in [40] and show good agreement between the computational and the experimental results.

The studies that dealt with the determination of the elastic constants of a material generally considered particular cases of RVE loading and, based on the particular strain and stress fields, determined some of these constants based on direct calculation [7,8,9,10,11,12,13]. Some of the methods obtained only lower and upper bounds of these constants, providing a relatively large range for these constants. In the case of this paper, several loading cases were considered and, based on them, elastic constants were considered using the least squares method. In this way, considering more loading cases (as many as possible) can minimize the error with which the elastic constants are determined. The accuracy with which these values are obtained should be higher than the calculation accuracy when considering a single loading state [60,61,62,63,64].

Other effects, such as thermal, humidity, etc., can be taken into account. But in this case, the variant corresponding to the considered case must be used in modeling. In this case, too, homogenization of the elastic coefficients can be carried out by averaging, but other physical parameters necessary for the studied model can also be obtained. So the method can be applied in any complex situation, but each time a corresponding model must be considered, which usually involves additional parameters.

## 5. Conclusions

The presented method gives us values of the engineering constants of a composite if these values are known for the components of the composite and if its topology and geometry are known. It is the case often encountered in practice that it is necessary to estimate the properties of a newly composed material. These values obtained are approximate and are affected by errors occurring in the use of FEM. In order to minimize these errors, the paper proposes the method of least squares, a mathematical method that provides the best estimate for a set of values obtained by calculating FEM. It is useful to consider as many load cases as possible to obtain the best estimate. The method has the advantage of using FEM, which has become a common method, verified by countless applications and frequently used in engineering. In this way, the sought values can be obtained with relative ease.

The main novelty is the application of the least squares method, which is a well-known method. With its help, several elastic constants of a material can be determined (in the case of isotropic materials, two; in the case of transversely isotropic materials, five; etc.). The precision in the case of this approximation method increases with the number of cases considered. For a model of a representative structure of the material, that model is made with finite elements for which the field of stresses and deformations is determined for several different loading cases. Based on these values, it is now possible to determine the engineering constants of the material using the least squares method, a simple and easy-to-apply method.

It is mentioned that the study of as many loading situations as possible should give us, according to the least squares theory, the best approximation of the elastic constants respecting the conditions presented in the paper. So the goal when applying the method is to perform as many calculations of the stress and strain field as possible, so as to obtain the most accurate results of the engineering constants sought.

## Figures and Tables

**Table 1:** Average strain in fiber for four loading cases.

Loading Case	${\bar{\varepsilon }}_{xx}$	${\bar{\varepsilon }}_{yy}$	${\bar{\varepsilon }}_{zz}$	${\bar{\varepsilon }}_{yz}$	${\bar{\varepsilon }}_{zx}$	${\bar{\varepsilon }}_{xy}$
1	3.60 × 10^−4^	−1.22 × 10^−4^	−1.22 × 10^−4^	−1.60 × 10^−13^	−1.01 × 10^−17^	−3.86 × 10^−17^
2	3.60 × 10^−4^	−1.22 × 10^−4^	−1.22 × 10^−4^	−8.54 × 10^−8^	−3.27 × 10^−9^	−5.43 × 10^−8^
3	3.11 × 10^−4^	−1.05 × 10^−4^	−1.05 × 10^−4^	−2.45 × 10^−4^	−7.56 × 10^−9^	2.41 × 10^−10^
4	1.79 × 10^−4^	−6.03 × 10^−5^	−6.02 × 10^−5^	−4.25 × 10^−4^	−1.46 × 10^−8^	2.33 × 10^−9^
5	−1.46 × 10^−6^	4.46 × 10^−7^	4.56 × 10^−7^	−4.91 × 10^−4^	−1.67 × 10^−8^	4.06 × 10^−11^

**Table 2:** Average strain in matrix for four loading cases.

Loading Case	${\bar{\varepsilon }}_{xx}$	${\bar{\varepsilon }}_{yy}$	${\bar{\varepsilon }}_{zz}$	${\bar{\varepsilon }}_{yz}$	${\bar{\varepsilon }}_{zx}$	${\bar{\varepsilon }}_{xy}$
1	3.60 × 10^−4^	−8.83 × 10^−5^	−8.83 × 10^−5^	−4.25 × 10^−17^	4.38 × 10^−11^	−1.11 × 10^−17^
2	3.60 × 10^−4^	−8.70 × 10^−5^	−9.15 × 10^−5^	−1.08 × 10^−7^	−2.74 × 10^−6^	−1.42 × 10^−7^
3	3.11 × 10^−4^	−7.95 × 10^−5^	−7.23 × 10^−5^	−2.48 × 10^−4^	4.86 × 10^−9^	−4.83 × 10^−10^
4	1.79 × 10^−4^	−4.56 × 10^−5^	−4.16 × 10^−5^	−4.29 × 10^−4^	8.71 × 10^−9^	−1.05 × 10^−9^
5	−1.33 × 10^−6^	3.69 × 10^−7^	2.32 × 10^−7^	−4.95 × 10^−4^	1.43 × 10^−8^	−1.53 × 10^−9^

**Table 3:** Average strain in RVE for four loading cases.

Loading Case	${\bar{\varepsilon }}_{xx}$	${\bar{\varepsilon }}_{yy}$	${\bar{\varepsilon }}_{zz}$	${\bar{\varepsilon }}_{yz}$	${\bar{\varepsilon }}_{zx}$	${\bar{\varepsilon }}_{xy}$
1	3.60 × 10^−4^	−1.09 × 10^−4^	−1.09 × 10^−4^	−2.84 × 10^−7^	−9.09 × 10^−7^	−2.84 × 10^−7^
2	3.60 × 10^−4^	−1.06 × 10^−4^	−1.08 × 10^−4^	−9.58 × 10^−8^	−1.25 × 10^−6^	−9.40 × 10^−8^
3	3.11 × 10^−4^	−9.33 × 10^−5^	−9.01 × 10^−5^	−2.46 × 10^−4^	−1.92 × 10^−9^	−8.84 × 10^−11^
4	1.79 × 10^−4^	−5.36 × 10^−5^	−5.18 × 10^−5^	−4.27 × 10^−4^	−4.01 × 10^−9^	7.93 × 10^−10^
5	−1.40 × 10^−6^	4.11 × 10^−7^	3.54 × 10^−7^	−4.93 × 10^−4^	−2.58 × 10^−9^	−6.73 × 10^−10^

**Table 4:** Average stress in fiber for four loading cases (in GPa).

Loading Case	${\bar{\sigma }}_{xx}$	${\bar{\sigma }}_{yy}$	${\bar{\sigma }}_{zz}$	${\bar{\sigma }}_{yz}$	${\bar{\sigma }}_{zx}$	${\bar{\sigma }}_{xy}$
1	3.14 × 10	4.71 × 10^−2^	4.71 × 10^−2^	−4.38 × 10^−8^	−5.65 × 10^−13^	−2.24 × 10^−12^
2	3.60 × 10^−4^	−1.22 × 10^−4^	−1.22 × 10^−4^	−8.54 × 10^−8^	−3.27 × 10^−9^	−5.43 × 10^−8^
3	3.11 × 10^−4^	−1.05 × 10^−4^	−1.05 × 10^−4^	−2.45 × 10^−4^	−7.56 × 10^−9^	2.41 × 10^−10^
4	1.79 × 10^−4^	−6.03 × 10^−5^	−6.02 × 10^−5^	−4.25 × 10^−4^	−1.46 × 10^−8^	2.33 × 10^−9^
5	−1.46 × 10^−6^	4.46 × 10^−7^	4.56 × 10^−7^	−4.91 × 10^−4^	−1.67 × 10^−8^	4.06 × 10^−11^

**Table 5:** Average stress in matrix for four loading cases (in GPa).

Loading Case	${\bar{\sigma }}_{xx}$	${\bar{\sigma }}_{yy}$	${\bar{\sigma }}_{zz}$	${\bar{\sigma }}_{yz}$	${\bar{\sigma }}_{zx}$	${\bar{\sigma }}_{xy}$
1	1.46 × 10^0^	−5.72 × 10^−2^	−5.72 × 10^−2^	1.08 × 10^−18^	−3.93 × 10^−14^	−1.60 × 10^−13^
2	3.60 × 10^−4^	−8.70 × 10^−5^	−9.15 × 10^−5^	−1.08 × 10^−7^	−2.74 × 10^−6^	−1.42 × 10^−7^
3	3.11 × 10^−4^	−7.95 × 10^−5^	−7.23 × 10^−5^	−2.48 × 10^−4^	4.86 × 10^−9^	−4.83 × 10^−10^
4	1.79 × 10^−4^	−4.56 × 10^−5^	−4.16 × 10^−5^	−4.29 × 10^−4^	8.71 × 10^−9^	−1.05 × 10^−9^
5	−1.33 × 10^−6^	3.69 × 10^−7^	2.32 × 10^−7^	−4.95 × 10^−4^	1.43 × 10^−8^	−1.53 × 10^−9^

**Table 6:** Average stress in RVE for four loading cases (in GPa).

Loading Case	${\bar{\sigma }}_{xx}$	${\bar{\sigma }}_{yy}$	${\bar{\sigma }}_{zz}$	${\bar{\sigma }}_{yz}$	${\bar{\sigma }}_{zx}$	${\bar{\sigma }}_{xy}$
1	1.78 × 10	−7.15 × 10^−3^	−7.14 × 10^−3^	−9.64 × 10^−4^	−3.08 × 10^−3^	−9.64 × 10^−4^
2	3.60 × 10^−4^	−1.06 × 10^−4^	−1.08 × 10^−4^	−9.58 × 10^−8^	−1.25 × 10^−6^	−9.40 × 10^−8^
3	3.11 × 10^−4^	−9.33 × 10^−5^	−9.01 × 10^−5^	−2.46 × 10^−4^	−1.92 × 10^−9^	−8.84 × 10^−11^
4	1.79 × 10^−4^	−5.36 × 10^−5^	−5.18 × 10^−5^	−4.27 × 10^−4^	−4.01 × 10^−9^	7.93 × 10^−10^
5	−1.40 × 10^−6^	4.11 × 10^−7^	3.54 × 10^−7^	−4.93 × 10^−4^	−2.58 × 10^−9^	−6.73 × 10^−10^

**Table 7:** Computed values of elastic moduli.

Modulus [MPa]	Matrix	Fiber	Average
$E_{11}$	4140.0	86,900.0	56,278.0
$E_{23}=E_{13}$	4140.0	86,899.0	12,741.0
$\nu_{1}$	0.34	0.22	0.28
$\nu_{23}$	0.34	0.22	0.31
$G_{23}$	1544.0	35,614.7	12,318.2
$K_{23}$	4827.4	63,597.7	27,953.2

## Data Availability

Data are contained within the article.

## Appendix A

$$\begin{array}{c} \left[\begin{array}{cccc} \sum_{i=1}^{n}{\bar{\varepsilon }}_{xx,i}^{2} & \sum_{i=1}^{n}{\bar{\varepsilon }}_{yy,i}{\bar{\varepsilon }}_{xx,i}+\sum_{i=1}^{n}{\bar{\varepsilon }}_{zz,i}{\bar{\varepsilon }}_{xx,i} & 0 & 0\\ \sum_{i=1}^{n}{\bar{\varepsilon }}_{xx,i}\left({\bar{\varepsilon }}_{yy,i}+{\bar{\varepsilon }}_{zz,i}\right) & \sum_{i=1}^{n}{\left({\bar{\varepsilon }}_{yy,i}+{\bar{\varepsilon }}_{zz,i}\right)}^{2}+2\sum_{1}^{n}{\bar{\varepsilon }}_{xx,i}^{2} & \sum_{1}^{n}{\bar{\varepsilon }}_{xx,i}{\bar{\varepsilon }}_{yy,i}+\sum_{1}^{n}{\bar{\varepsilon }}_{xx,i}{\bar{\varepsilon }}_{zz,i} & \sum_{1}^{n}{\bar{\varepsilon }}_{xx,i}{\bar{\varepsilon }}_{zz,i}+\sum_{1}^{n}{\bar{\varepsilon }}_{xx,i}{\bar{\varepsilon }}_{yy,i}\\ 0 & \sum_{1}^{2}{\bar{\varepsilon }}_{xx,i}{\bar{\varepsilon }}_{yy,i}+\sum_{1}^{n}{\bar{\varepsilon }}_{xx,i}{\bar{\varepsilon }}_{zz,i} & \sum_{1}^{n}{\bar{\varepsilon }}_{yy,1}^{2}+\sum_{1}^{n}{\bar{\varepsilon }}_{zz,i}^{2}+2\sum_{1}^{n}{\bar{\varepsilon }}_{yz,i}^{2} & 2\sum_{1}^{n}{\bar{\varepsilon }}_{zz,i}{\bar{\varepsilon }}_{yy,i}+\sum_{1}^{n}{\bar{\varepsilon }}_{yz,i}^{2}\\ 0 & \sum_{i}^{n}{\bar{\varepsilon }}_{xx,i}{\bar{\varepsilon }}_{zz,i}+\sum_{1}^{n}{\bar{\varepsilon }}_{xx,i}{\bar{\varepsilon }}_{yy,i} & 2\sum_{1}^{n}{\bar{\varepsilon }}_{zz,i}{\bar{\varepsilon }}_{yy,i}+\sum_{1}^{n}{\bar{\varepsilon }}_{yz,i}^{2} & \sum_{1}^{n}{\bar{\varepsilon }}_{zz,i}^{2}+\sum_{1}^{n}{\bar{\varepsilon }}_{yy,i}^{2}+\sum_{1}^{n}{\bar{\varepsilon }}_{yz,i}^{2} \end{array}\right]\left\{\begin{array}{c} C_{11}\\ C_{12}\\ C_{22}\\ C_{23} \end{array}\right\}\\ =\left\{\begin{array}{c} \sum_{i=1}^{n}{\bar{\sigma }}_{xx,i}{\bar{\varepsilon }}_{xx,i}\\ \sum_{i=1}^{n}{\bar{\sigma }}_{xx,i}\left({\bar{\varepsilon }}_{yy,i}+{\bar{\varepsilon }}_{zz,i}\right)+\sum_{1}^{n}{\bar{\sigma }}_{yy,i}{\bar{\varepsilon }}_{xx,i}+\sum_{1}^{n}{\bar{\sigma }}_{zz,i}{\bar{\varepsilon }}_{xx,i}\\ \sum_{i}^{n}{\bar{\sigma }}_{yy,i}{\bar{\varepsilon }}_{yy,i}+\sum_{1}^{n}{\bar{\sigma }}_{zz,i}{\bar{\varepsilon }}_{zz,i}+2\sum_{1}^{n}{\bar{\sigma }}_{yz,i}{\bar{\varepsilon }}_{yz,i}\\ \sum_{1}^{n}{\bar{\sigma }}_{yy,i}{\bar{\varepsilon }}_{zz,i}+\sum_{1}^{n}{\bar{\sigma }}_{zz,i}{\bar{\varepsilon }}_{yy,i}+\sum_{1}^{n}{\bar{\sigma }}_{yz,i}{\bar{\varepsilon }}_{yz,i} \end{array}\right\}, \end{array}$$

## References

[1] Katouzian M. (1995). On the Effect of Tempeature on Creep Behavior of Neat and Carbon Fiber Reinforced PEEK and Epoxy—A Micromechanical Approach. Ph.D. Thesis.

[2] Findley W.N., Khosla G. (1955). Application of the Superposition Principle and Theories of Mechanical Equation of State, Strain, and Time Hardening to Creep of Plastics under Changing Loads. J. Appl. Phys..

[3] Hashin Z. (1965). On Elastic Behavior of Fibre Reinforced Materials of Arbitrary Transverse Phase Geometry. J. Mech. Phys. Solids.

[4] Hashin Z., Shtrikman S. (1963). A Variational Approach to the Theory of the Elastic Behavior of Multiphase Materials. J. Mech. Phys. Solids.

[5] Hashin Z., Rosen B.W. (1964). The Elastic Moduli of Fiber-Reinforced Materials. J. Appl. Mech..

[6] Bowles D.E., Griffin O.H. (1991). Micromechanics Analysis of Space Simulated Thermal Stresses in Composites. Part I: Theory and Unidirectional Laminates. J. Reinf. Plast. Compos..

[7] Zhao Y.H., Weng G.J. (1990). Effective Elastic Moduli of Ribbon-Reinforced Composites. J. Appl. Mech..

[8] Hill R. (1964). Theory of Mechanical Properties of Fiber-strengthened Materials: I Elastic Behavior. J. Mech. Phys. Solids.

[9] Hill R. (1964). Theory of Mechanical Properties of Fiber-strengthened Materials: II Inelastic Behavior. J. Mech. Phys. Solids.

[10] Hill R. (1965). Theory of Mechanical Properties of Fiber-strengthened Materials: III Self-Consistent Model. J. Mech. Phys. Solids.

[11] Hill R. (1965). Continuum Micro-Mechanics of Elastoplastic Polycrystals. J. Mech. Phys. Solids.

[12] Aboudi J. (1990). Micromechanical characterization of the non-linear viscoelastic behavior of resin matrix composites. Compos. Sci. Technol..

[13] Aboudi J. (1991). Mechanics of Composite Materials—A Unified Micromechanical Approach.

[14] Othman M.I.A., Fekry M., Marin M. (2020). Plane waves in generalized magneto-thermo-viscoelastic medium with voids under the effect of initial stress and laser pulse heating. Struct. Eng. Mech..

[15] Marin M., Hobiny A., Abbas I. (2021). The Effects of Fractional Time Derivatives in Porothermoelastic Materials Using Finite Element Method. Mathematics.

[16] Vlase S., Teodorescu-Draghicescu H., Motoc D.L., Scutaru M.L., Serbina L., Calin M.R. (2011). Behavior of Multiphase Fiber-Reinforced Polymers Under Short Time Cyclic Loading. Optoelectron. Adv. Mater. Rapid Commun..

[17] Abbas I., Hobiny A., Marin M. (2020). Photo-thermal interactions in a semi-conductor material with cylindrical cavities and variable thermal conductivity. J. Taibah Univ. Sci..

[18] Bratu P., Dobrescu C., Nitu M.C. (2023). Dynamic Response Control of Linear Viscoelastic Materials as Resonant Composite Rheological Models. Rom. J. Acoust. Vib..

[19] Niculiţă C., Vlase S., Bencze A., Mihălcică M., Calin M.R., Serbina L. (2011). Optimum stacking in a multiply laminate used for the skin of adaptive wings. Optoelectron. Adv. Mater. Rapid Commun..

[20] Katouzian M., Vlase S., Calin M.R. (2011). Experimental procedures to determine the viscoelastic parameters of laminated composites. J. Optoelectron. Adv. Mater..

[21] Abo-Dahab S.M., Abouelregal A.E., Marin M. (2020). Generalized Thermoelastic Functionally Graded on a Thin Slim Strip Non-Gaussian Laser Beam. Symmetry.

[22] Fliegener S., Hohe J. (2020). An anisotropic creep model for continuously and discontinuously fiber reinforced thermoplastics. Compos. Sci. Technol..

[23] Xu B., Xu W., Guo F. (2020). Creep behavior due to interface diffusion in unidirectional fiber-reinforced metal matrix composites under general loading conditions: A micromechanics analysis. Acta Mech..

[24] Lal H.M.M., Xian G.-J., Thomas S., Zhang L., Zhang Z., Wang H. (2020). Experimental Study on the Flexural Creep Behaviors of Pultruded Unidirectional Carbon/Glass Fiber-Reinforced Hybrid Bars. Materials.

[25] Wang Z., Smith D.E. (2019). Numerical analysis on viscoelastic creep responses of aligned short fiber reinforced composites. Compos. Struct..

[26] Fattahi A.M., Mondali M. (2014). Theoretical study of stress transfer in platelet reinforced composites. J. Theor. Appl. Mech..

[27] Fattahi A.M., Moaddab E., Bibishahrbanoei N. (2015). Thermo-mechanical stress analysis in platelet reinforced composites with bonded and debonded platelet end. J. Mech. Sci. Technol..

[28] Tebeta R.T., Fattahi A.M., Ahmed N.A. (2021). Experimental and numerical study on HDPE/SWCNT nanocomposite elastic properties considering the processing techniques effect. Microsyst. Technol..

[29] Selmi A., Friebel C., Doghri I., Hassis H. (2007). Prediction of the elastic properties of single walled carbon nanotube reinforced polymers: A comparative study of several micromechanical models. Compos. Sci. Technol..

[30] Stanciu A., Teodorescu-Drǎghicescu H., Vlase S., Scutaru M.L., Cǎlin M.R. (2012). Mechanical behavior of CSM450 and RT800 laminates subjected to four-point bend tests. Optoelectron. Adv. Mater. Rapid Commun..

[31] Tran A.B., Yvonnet J., He Q.C., Toulemonde C., Sanahuja J. (2011). A simple computational homogenization method for structures made of linear heterogeneous viscoelastic materials. Comput. Methods Appl. Mech. Eng..

[32] Katouzian M., Vlase S., Scutaru M.L. (2021). Finite Element Method-Based Simulation Creep Behavior of Viscoelastic Carbon-Fiber Composite. Polymers.

[33] Fung Y.C. (1965). Fundamentals of Solid Mechanics.

[34] Schapery R.A. (1967). Stress Analysis of Viscoelastic Composite Materials. J. Compos. Mater..

[35] Morris D.H., Yeow Y.T., Brinson H.F. (1979). The Viscoelastic Behavior of the Principal Compliance Matrix of Unidirectional Graphite Epoxy Composite.

[36] Huang Z.-M. (2021). Constitutive relation, deformation, failure and strength of composites reinforced with continuous/short fibers or particles. Compos. Struct..

[37] Huang Z.-M. (2019). A micromechanics approach to stiffness and strength of unidirectional composites. J. Reinf. Plastics Comp..

[38] Hinton M.J., Kaddour A.S., Soden P.D. (2004). The world-wide failure exercise: Its origin, concept and content. The World-Wide Failure Exercise.

[39] Ahmadi M., Ansari R., Hassanzadeh-Aghdam M.K. (2023). Micromechanical finite element analysis of Young’s modulus, yield strength and thermal expansion coefficient of nano-sized ceramic particle/metal matrix nanocomposites. J. Braz. Soc. Mech. Sci. Eng..

[40] Oz F.E. (2022). Computational examination of the effect of voids on the mechanical response of composites with emphasize on the cure hardening behavior. Mech. Adv. Mater. Struct..

[41] Christofi I., Hadjiloizi D.A., Georgiades A.V. (2022). Dynamic micromechanical model for smart composite and reinforced shells. Zamm-Z. Fur Angew. Math. Und Mech..

[42] Gupta M., Ray M.C., Kundalwal S.I. (2021). Dynamic modelling and analysis of smart carbon nanotube-based hybrid composite beams: Analytical and finite element study. Proc. Inst. Mech. Engineers. Part L-J. Mater. Des. Appl..

[43] Bratu P., Vlase S., Dragan N., Vasile O., Itu C., Nitu C.M., Toderita A. (2022). Modal Analysis of the Inertial Platform of the Laser ELI-NP Facility in Magurele-Bucharest. Rom. J. Acoust. Vib..

[44] Mishra V.N., Sarangi S.K. (2023). A Numerical Model for the Effective Damping Properties of Unidirectional Fiber-Reinforced Composites. Mech. Compos. Mater..

[45] Qin F.P., Lu F.C., Huang L. (2023). Numerical simulation and experimental validation of ratchetting deformation of short fiber-reinforced polymer composites. Compos. Part B Eng..

[46] Vázquez J.M.C., Wu L., Noels L. (2023). A micromechanical mean-field homogenization surrogate for the stochastic multiscale analysis of composite materials failure. Int. J. Numer. Methods Eng..

[47] Manchiraju V.N.M., Bhagat A.R., Dwivedi V.K. (2023). Estimation of Elastic Constants Using Numerical Methods and Their Validation Through Experimental Results for Unidirectional Carbon/Carbon Composite Materials. Jpn. J. Metrol. Soc. India.

[48] de Morais A.B. (2000). Transverse moduli of continuous-fibre-reinforced polymers. Compos. Sci. Technol..

[49] Fuchs M.B., Paley M., Miroshny E. (1998). Evaluation of failure criteria for fiber composites using finite element micromechanics. J. Compos. Mater..

[50] Astaraki S., Zamani E., Mohamadipoor R. (2023). Determination of mechanical properties of nanocomposites reinforced with spherical silica nanoparticles using experiments, micromechanical model and finite elements method. J. Compos. Mater..

[51] Scutaru M.L., Vlase S., Marin M., Modrea A. (2020). New analytical method based on dynamic response of planar mechanical elastic systems. Bound. Value Probl..

[52] Li Y., Li Y.Q. (2019). Evaluation of elastic properties of fiber reinforced concrete with homogenization theory and finite element simulation. Constr. Build. Mater..

[53] Nguyen A.V., Nguyen T.C. Homogenization of Viscoelastic Composite Reinforced Woven Flax Fibers. Proceedings of the 11th Joint Canada-Japan Workshop on Composites/1st Joint Canada-Japan-Vietnam Workshop on Composites.

[54] Matsuda T., Ohno N. (2011). Predicting the elastic-viscoplastic and creep behaviour of polymer matrix composites using the homogenization theory. Creep and Fatigue in Polymer Matrix Composites.

[55] Tian W.L., Qi L.H., Jing Z. (2016). Numerical simulation on elastic properties of short-fiber-reinforced metal matrix composites: Effect of fiber orientation. Compos. Struct..

[56] Zhu T.L., Wang Z. (2023). Research and application prospect of short carbon fiber reinforced ceramic composites. J. Eur. Ceram. Soc..

[57] Griffith W.I. (1979). The Accelerated Characterization of Viscoelatic Composite Materials.

[58] Teodorescu-Draghicescu H., Stanciu A., Vlase S., Scutaru L., Calin M.R., Serbina L. (2011). Finite Element Method Analysis of Some Fibre-Reinforced Composite Laminates. Optoelectron. Adv. Mater. Rapid Commun..

[59] Sá M.F., Gomes A., Correia J., Silvestre N. (2011). Creep behavior of pultruded GFRP elements—Part 1: Literature review and experimental study. Compos. Struct..

[60] Wu J., Zhu Y., Li C. (2023). Experimental Investigation of Fatigue Capacity of Bending-Anchored CFRP Cables. Polymers.

[61] Alhoubi Y., Mahaini Z., Abed F. (2022). The Flexural Performance of BFRP-Reinforced UHPC Beams Compared to Steel and GFRP-Reinforced Beams. Sustainability.

[62] Xian G., Guo R., Li C. (2022). Combined effects of sustained bending loading, water immersion and fiber hybrid mode on the mechanical properties of carbon/glass fiber reinforced polymer composite. Compos. Struct..

[63] Kamiński M., Ostrowski P. (2021). Homogenization of heat transfer in fibrous composite with stochastic interface defects. Compos. Struct..

[64] Ostoja-Starzewski M., Sheng P.Y., Jasiuk I. (1997). Damage patterns and constitutive response of random matrix-inclusion composites. Eng. Fract. Mech..

